# SEQEL: a tool for biological sequence manipulation in Emacs

**DOI:** 10.1093/bioadv/vbab019

**Published:** 2021-11-17

**Authors:** Zhenjiang Zech Xu, Hui Lu

**Affiliations:** State Key Laboratory of Food Science and Technology, Nanchang University, Nanchang, Jiangxi 330029, China

## Abstract

**Summary:**

Sequences are arguably the most common biological data. An easy-to-use tool can greatly facilitate daily manipulation and analysis of biological sequences. Here, we present SEQEL, a tool providing a convenient environment for editing, formatting and rendering of DNA, RNA and protein sequences. This is accomplished by extending the commonly used text editor, Emacs, which is available for Windows, Linux and Mac OS.

**Availability and Implementation:**

The unit tested ELISP source code for seqel is freely available from https://github.com/rnaer/seqel along with documentation.

**Contact:**

zhenjiang.xu@gmail.com

## 1 Introduction

Since the first determination of biological sequence, the B chain of insulin, by Sanger in 1951 (Sanger and Tuppy, 1951), the past decades have witnessed tremendous advances of sequencing technologies, especially for nucleic acid sequences, moving from labor-intensive short-oligonucleotide sequencing to massive parallel high-throughput sequencing. This has resulted in an unprecedented proliferation of sequence data and led to the development of efficient computational algorithms for sequence analysis to compare, align, index, search and assemble biological sequences (Edgar and Batzoglou, 2006; Nagarajan and Pop, 2013). These tools greatly automate the processes of generating hypotheses and mining insights from raw sequence data.

However, most computational tools have their own presumptions or limitations. Manual inspection is still frequently required to check or verify the input or output sequence data. Although tools are available for specific application, including alignment editors (Griffiths-Jones, 2005; Larsson, 2014), sequence manipulation (Stothard, 2000; Sun, 2018), plasmid drawing (Dong *et al.*, 2004), etc., a lightweight, general-purpose tool that is natively integrated with a text editor is still missing for rapid, easy biological sequence examination. We developed an Emacs extension, SEQEL, to visualize, edit and format biological sequences so that users can effectively examine and manipulate sequence files without having to leave their familiar text/code editor and turn to another software for simple operations.

## 2 Features

**Fig. 1.**
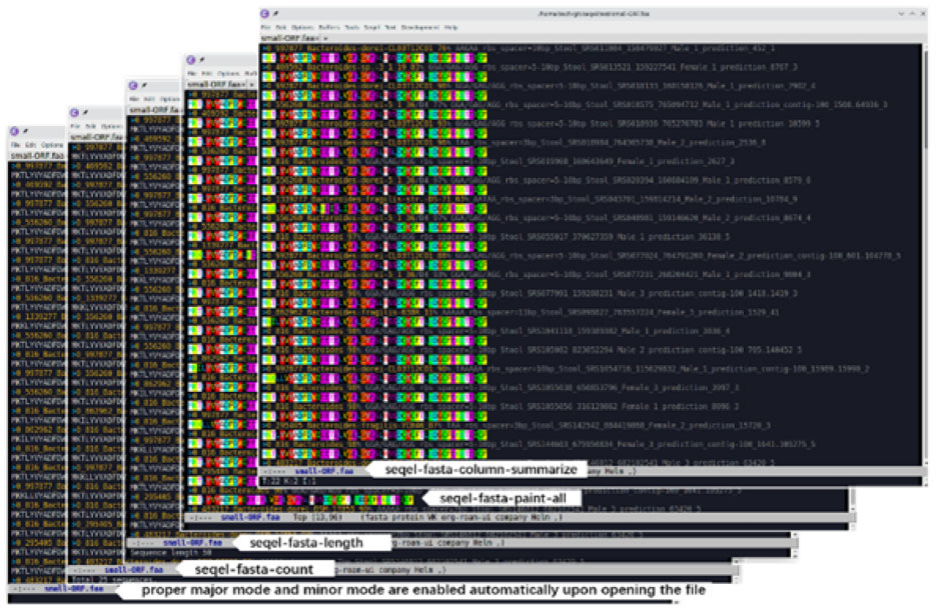
A quick inspection of putative small proteins identified in human associated metagenomes using SEQEL

SEQEL is written in native Emacs Lisp (ELISP) and easily installed as an Emacs extension using Emacs’ built-in package management system. SEQEL detects Fasta or Genbank file formats based on their file suffices and automatically enables proper file major mode for font locking (i.e. syntax highlighting) and functionality loading in Emacs. Currently, SEQEL supports nine simple but effective functionalities: viewing, editing, summarizing and motif searching. For viewing, SEQEL allows users to navigate sequences, jumping from one sequence record to another with convenient and customizable keyboard shortcuts. And sequences can be colorized according to different residues or by other user-defined rules. For editing, users can easily delete a whole sequence record, convert nucleotide sequences to reverse complements, translate nucleotide sequences to proteins and convert Genbank format to Fasta. SEQEL also provides summary information including sequence counts, sequence length, residue frequencies and protein molecular weight. Taking advantage of Emacs powerful incremental search function, SEQEL allows case-sensitive or insensitive motif search (supporting IUPAC code degeneracy) and ignores the possible whitespaces, gaps, or other nonresidue characters (e.g. ‘-‘, ‘*’) in the middle of matching hits. Additionally, for aligned sequence file, SEQEL also provides column-wise operations to insert, delete, colorize or summarize the residues in the same position across all aligned sequences (Fig. 1). We provide two simple use cases in the following, showing how SEQEL facilitate rapid and easy sequence examination in native Emacs, without turning to a third-party heavy-lifting tool.

Use case 1: Recently a study identified putative small proteins (no more than 50 amino acids in length) in human-associated metagenomes (Sberro *et al.*, 2019). With Emacs SEQEL, we opened the first family of these small proteins provided in the supplementary material of the article. The fasta major mode and protein minor mode were automatically enabled with sequence header colored. Running ‘seqel-fasta-count’ and ‘seqel-fasta-length’ showed that there were 25 proteins in this family and they were 50 amino acids long. These sequences looked very similar and we painted the amino acids to help check quickly with naked eyes if there were different residues differing across sequences. After spotting the difference in the third column of residues, we ran ‘seqel-fasta-column-summarize’ to get a summary count of how many of each residue is in this column (Fig. 1).

**Fig. 2.**
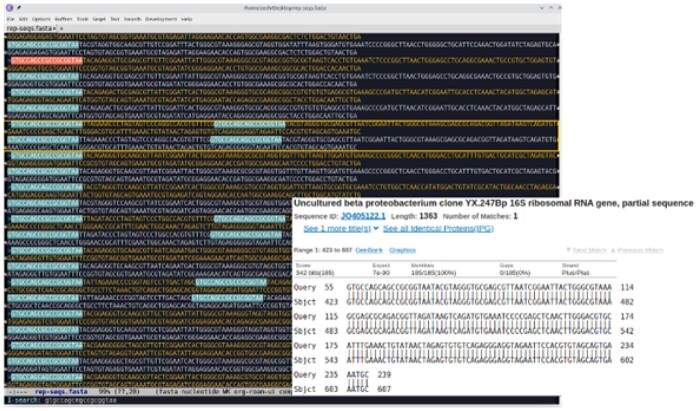
SEQEL searched the primer containing IUPAC degenerate codes and found dubious sequences that had two forward primers (as shown in the yellow box)

Use case 2: For a meta-analysis of the gastric microbiome, we downloaded a public data of 16S rRNA amplicon sequencing from NCBI PRJNA375772 and reanalyzed it with QIIME2 (Bolyen *et al.*, 2019). We found that this dataset had an unusually large number (>100) of different *Helicobacter pylori* amplicon sequence variants (ASVs) (Amnon *et al.*, 2017) and raised our doubt about data quality. With Emacs SEQEL, we opened the amplicon sequence file for a quick inspection. We first checked if there were problems on sequencing adaptors or primers, which is often the culprit in the public amplicon sequencing data. We did a simple search in the file using the primer listed in the original publication (forward: 5′-GTGCCAGCMGCCGCGGTAA-3′ and reverse: 5′- GGACTACHVGGGTWTCTAAT-3′) (Coker *et al.*, 2018). SEQEL supports the ambiguous degenerate code in the primer during pattern search and identified that there were sequences containing two forward primers (Fig. 2). We took the sequences and blasted against NCBI nucleotide database. The alignment of the best blast hit showed that the nucleotides up to the second forward primer of those sequences did not match 16S rRNA and were likely artifacts resulting from sequencing library preparation.

In summary, SEQEL has the following advantages:

- It does not force users to leave the text editor and launch another software for simple sequence inspection and manipulations.
- It inherits core functionalities and general merits of Emacs: convenient file handling and buffer operations (e.g. open compressed fasta files without explicitly extraction), access to remote sequence files via Transparent Remote Access, Multiple Protocols mode (TRAMP), long undo and redo chain, incremental search, font locking and high customizability.
- It conforms to the same Emacs interface that is already familiar to a large user base of Emacs.
- It runs on most operating systems as Emacs is available for Windows and UNIX-derived platforms.
- It is unit tested to ensure the code quality and verify the expected behavior of each functionality (including font highlighting and colorization).

## 3 Conclusions

The SEQEL package provides a convenient tool that supports rapid, easy manipulation of sequence data in the widely used, multiplatform text editor, Emacs. The implementation of SEQEL as an Emacs extension makes it readily adaptable by the large user base of Emacs and allows advanced users to customize as they would like. In conclusion, we believe that SEQEL can effectively simplify the workflow of manual sequence inspection and manipulation.
